# Synthetic promoter design in *Escherichia coli* based on multinomial diffusion model

**DOI:** 10.1016/j.isci.2024.111207

**Published:** 2024-10-18

**Authors:** Qixiu Du, May Nee Poon, Xiaocheng Zeng, Pengcheng Zhang, Zheng Wei, Haochen Wang, Ye Wang, Lei Wei, Xiaowo Wang

**Affiliations:** 1 Ministry of Education Key Laboratory of Bioinformatics, Center for Synthetic and Systems Biology, Bioinformatics Division, Beijing National Research Center for Information Science and Technology, Department of Automation, Tsinghua University, Beijing 100084, China

**Keywords:** Gene process, *In silico* biology, Biological constraints, Artificial intelligence

## Abstract

Generative design of promoters has enhanced the efficiency of *de novo* creation of functional sequences. Though several deep generative models have been employed in biological sequence generation, including variational autoencoder (VAE) or Wasserstein generative adversarial network (WGAN), these models might struggle with mode collapse and low sample diversity. In this study, we introduce the multinomial diffusion model (MDM) for promoter sequence design and propose a structured set of criteria for effectively comparing the performance of generative models. *In silico* experiments demonstrate that MDM outperforms existing generative AI approaches. MDM demonstrates superior performance in various computational evaluations, remains robust during the training process, and exhibits a strong ability in capturing weak signals. In addition, we experimentally validated that the majority of our model designed promoters have expression activities *in vivo*, indicating the practicality and potential of MDM for bioengineering.

## Highlights

• We introduced MDM, a diffusion model for efficient promoter design in *Escherichia coli*
• We organized metrics and benchmarks to evaluate generative AI for sequence design
• MDM performs better across multiple computational evaluations
• MDM generates promoters with high *in vivo* validation performance

## Graphical abstract

**Figure:**
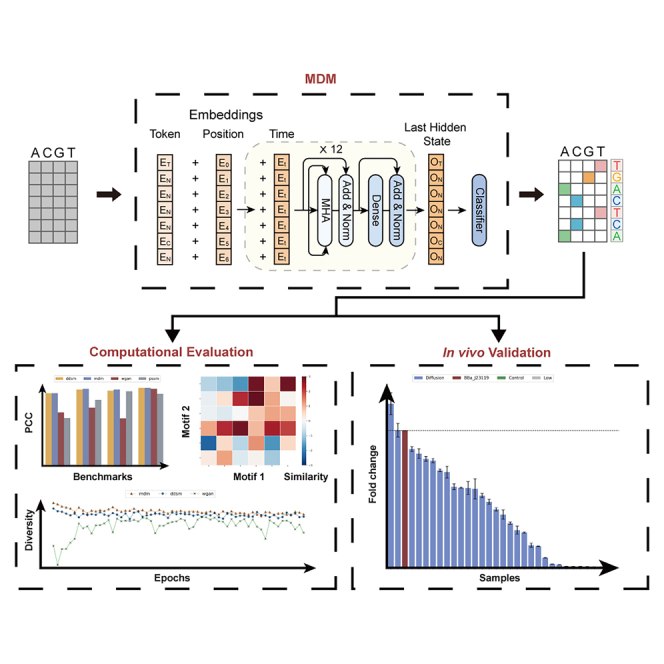

## Introduction

Promoter is a genomic region where transcription is initiated. Regulatory proteins such as transcription factors (TFs) bind to specific motifs to regulate gene activity. The rational design of promoter is an important task in synthetic biology, gene therapy, and various relevant domains.^1^^,^^2^ However, due to the complex protein-promoter interactions, designing promoters with desirable function and properties based solely on known biological knowledge (e.g., TF binding sequence motif) is tough. In addition, the potential sequence space is large, for a 50 base pairs (bp) promoter, the number of all possible sequences is $4^{50}$. Therefore, computational algorithms for sequence optimization remain challenging.

In recent years, deep learning models have made great achievements in various biological fields, including disease detection, precision medicine, enzyme engineering, and biosystem design.^3^^,^^4^ The exceptionally strong ability of feature representation has led researchers to utilize deep learning models to design biological sequences including DNA (e.g., promoters^5^^,^^6^^,^^7^ and enhancers^8^^,^^9^), RNA (e.g., UTRs^10^^,^^11^), or proteins^12^^,^^13^^,^^14^ with desired properties. Several studies have implemented AI-assisted promoter design by using a pre-trained predictor to introduce mutations in natural sequences and identify potential variants.^5^^,^^15^^,^^16^ These strategies control the edit distance between generated and the natural sequences, which limits the exploration of potential functional space. Another approach in promoter design is based on deep generative model. The current mainstream generative methods for promoter design are often based on generative adversarial networks (GANs).^6^^,^^7^^,^^13^^,^^17^ For instance, we previously proposed a model based on Wasserstein generative adversarial network (WGAN)^6^ for *de novo E. coli* promoter design, and achieved good performance in biological experiments. However, existing GANs have many drawbacks, including difficulty in converging, mode collapse,^18^ and unstable generation performance. These bring challenges in practical application, without determining relevant parameters through series of pre-experiments, promoters generated by GANs may not have the desired functionality and performance. Compared to GAN, the diffusion model can avoid these drawbacks and has been widely utilized in computer vision, natural language processing, and bioinformatics. Recently there have been some efforts to apply diffusion models to novel DNA design,^19^^,^^20^^,^^21^ and advanced diffusion models like Dirichlet diffusion score model (DDSM) and continuous diffusion model with UNet (PromoDiff) have been proposed. However, the superiority of the diffusion model still lacks thorough computational analysis and has not been fully explored in biological validation, this prompts us to evaluate these approaches and select a more effective generative model.

**Figure 1:**
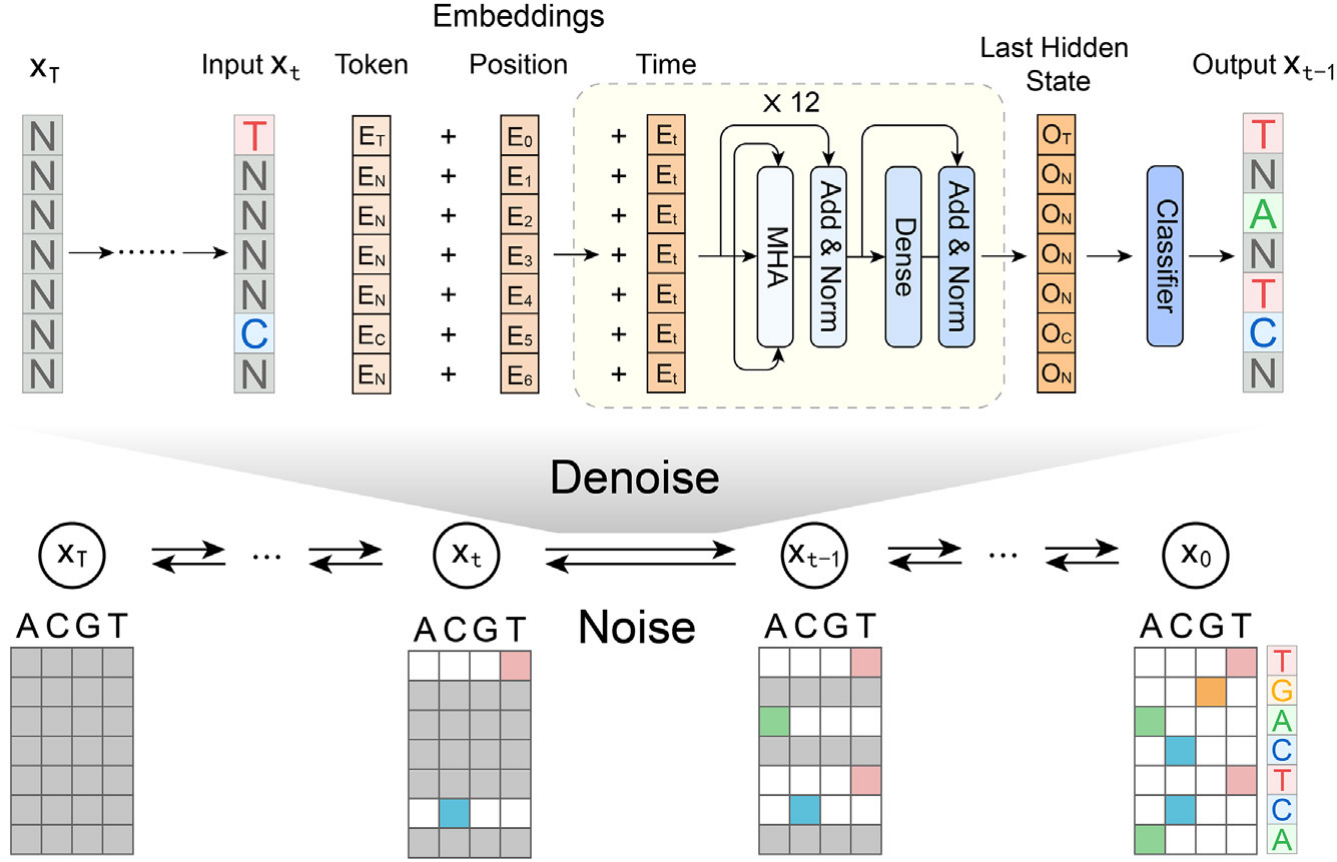
The noising and denoising process of multinomial diffusion model In the noise-adding process, a DNA sequence is step-by-step contaminated with noise until it becomes a random sequence. Here, the four base distribution probabilities are averaged. During the denoising process, a sequence is sampled from the random space and then step-by-step purged of noise through multiple transformer layers, until it adheres to the biological feature distribution. Here, N or a gray box represents an undetermined position. As the denoising process progresses, all undetermined bases will eventually transform into known bases.

In this paper, we proposed a generative AI framework that incorporates a multinomial diffusion model (MDM)^22^ for promoter design. The detailed framework of MDM is illustrated in Figure 1. We also developed a series of organized data quality assessment criteria for generative models, integrating multiple knowledge-based and data-driven approaches. Our results indicated that MDM can precisely capture natural patterns such as core motifs and k-mer frequency. Additionally, our simulations suggested that MDM does not suffer from mode collapse and can effectively decouple mixed weak signals. Finally, we conducted a reporter assay to validate the activity of MDM generated promoters. MDM generated promoter successfully drive the expression of reporter gene, while some of our designed promoters even show higher expression levels compared with very strong natural promoter BBa_J23119.^23^ Overall, both the computational and experimental results indicate that our framework is significantly effective for *E. coli* promoter design.

## Results

### AI-assisted promoter design process, benchmarks, and computational evaluation metrics

The generative AI framework for promoter design involves three steps. Firstly, we train the generative model p on dataset $X_{0}$, consisting of multiple *E. coli* sequences. Then, we train the predictive model q on paired dataset $\left(X_{0},Y_{0}\right)$, where $Y_{0}$ symbols the corresponding expression levels of $X_{0}$. Finally, we utilize the trained generator p to generate novel promoters $X_{new}$ and utilize the pretrained predictor q to predict the expression levels $Y_{new}$ of novel samples. The equation for training process can be formulated as:

$$\hat{\theta }=\arg\underset{\theta }{\max}\;p_{\theta }\left(X_{0}|z\right)$$

(Equation 1) $$\hat{\phi }=\arg\underset{\phi }{\max\;}q_{\phi }\left(Y_{0}|X_{0}\right)$$

$$X_{new}=p_{\hat{\theta }}\left(z\right),Y_{new}=q_{\hat{\phi }}\left(X_{new}\right)$$

Here, z follows a hidden latent distribution for generation, e.g., uniform distribution or normal distribution, θ symbols the trainable parameters of the generator p, and ϕ symbols the parameters of the predictor q. After the training process, new sequences can be generated from $p_{\hat{\theta }}$. There is currently a range of research on machine learning-based promoter design that can fit into this design paradigm.^24^^,^^25^ In this research, we employ MDM model for generation and CNN model for prediction. We have provided detailed information about the generative model and the predictive model in the STAR Methods section, including the model structures, the training processes, the sampling scale for the generator, and the parameters for the predictor.

Throughout this work, we evaluate the performance of generative models on previously validated datasets from various research groups, which we refer to as “benchmarks” in this paper. The core dataset in this paper originates from Thomason et al.,^26^ which contains 14,098 experimentally identified promoters in the *E. coli* K12 MG1655 genome. These promoters, which are 50 bp σ70 promoters, have corresponding gene expression levels measured by differential RNA sequencing (dRNA-seq) and can be distinguished based on essential motifs, particularly the ˗10 and ˗35 regions. This dataset has been validated and demonstrated robustness as a benchmark.^6^ To prevent bias from high internal similarity in training data, we checked sequence similarity and ensured most were unique in at least 15 of 50 bp (Figure S1). Additionally, we include three datasets for computational validation, which vary in sequence length, regulatory regions, and species. These datasets include: (1) dataset from Johns et al.,^27^ where they collected cross-species datasets across different genetic backgrounds and environmental contexts, and each sequence is sampled from the 165 bp immediately upstream of annotated start codons. We selected 13,972 *E. coli* sequences with expression levels quantified by RNA-seq, featuring a more complex *cis*-regulatory grammars (2) dataset from Vaishnav et al.,^15^ where they collected more than 30 million random sequences that were transformed into promoter regions in the *Saccharomyces cerevisiae*, cultured in YPD medium, and had their expression levels measured through a gigantic parallel reporter assay (GPRA). The total length of the promoter sequences is 110 bp, and we removed the 30-bp flanking sequences to isolate the 80-bp core promoter regions, resulting in a selection of 102,954 sequences (3) dataset from Zrimec et al.^7^ that involves the measurement of the performance of model-generated sequences, where each sequence 1,000 bp in length, encompassing promoters, coding regions, terminators, and untranslated regions. The expression levels of these sequences were measured using RNA-seq in the *Saccharomyces cerevisiae*, and we selected 4,238 sequences for validation.

In this work, we provide a series of organized, empirically derived computational validation metrics to evaluate model performances. We firstly discussed the capability of different generative AIs by comparing their corresponding generated sequences on several knowledge-based metrics (e.g., enrichment of core motifs) and data-driven metrics (e.g., the consistency of the frequency of occurrence for each 6-mer fragment with that in natural sequences). We subsequently assessed the stability and robustness of the models by analyzing all sequences sampled over a 60-epoch training process, based on multiple criteria (e.g., the level of data diversity in each round of generation). We then evaluated the signal decoupling ability of the models by designing a simulation dataset consisting of 36 mixed motif combinations, and compared the maintenance levels of these grammars in the model-generated sequences. Detailed computational validation processes are provided in the latter sections and STAR Methods.

### MDM generates sequences capturing essential biological features of high quality

The consistent biological feature distribution of AI-designed promoters with natural sequences is a pivotal criterion for guaranteeing their adaptability to cellular environment. Here, we compared our MDM with previous deep generative models designed for DNA sequences, including DDSM,^19^ PromoDiff,^20^ and WGAN.^6^ All training sequences are represented using one-hot encoding. Both MDM and DDSM are set with the default learning rate, their training epochs are both set to 100, and their parameters both vary slightly differently for each benchmark. The PromoDiff model is directly loaded from the pretrained model weights provided by Wang et al.^20^ In addition, the WGAN model is set with 12 training epochs, since higher training epochs might lead to mode collapse and unstable output,^6^ and other parameters are set as default. We also sampled from the position-specific scoring matrix (PSSM) of natural sequences to establish a control group. Here, the implementations of DDSM and PromoDiff are from their original publications, while the implementations of MDM and WGAN are directly by the GPro package,^28^ the package we previously proposed for efficiently designing sequences with generative AIs (STAR Methods).

To systematically assess the quality of sequences produced by various generative AI models, we consider not only their alignment with preexisting knowledge but also their performance against data-driven metrics. We have employed three knowledge-based metrics to separately assess the designed promoters, evaluating their conservation with respect to functional motifs, the distribution patterns among motifs, and their similarity to natural genomic sequences. Furthermore, to systematically evaluate the model’s performance across a broader range of application scenarios, including DNA sequences of different species, lengths, and functional regions, we integrated datasets from four distinct data sources, comprising *E. coli* and yeast sequences of various lengths that span different functional regions. We assessed the designed sequences against these data sources using two data-driven metrics: GC content and k-mer similarity, and evaluated the diversity of the obtained data across multiple random seeds.

**Figure 2:**
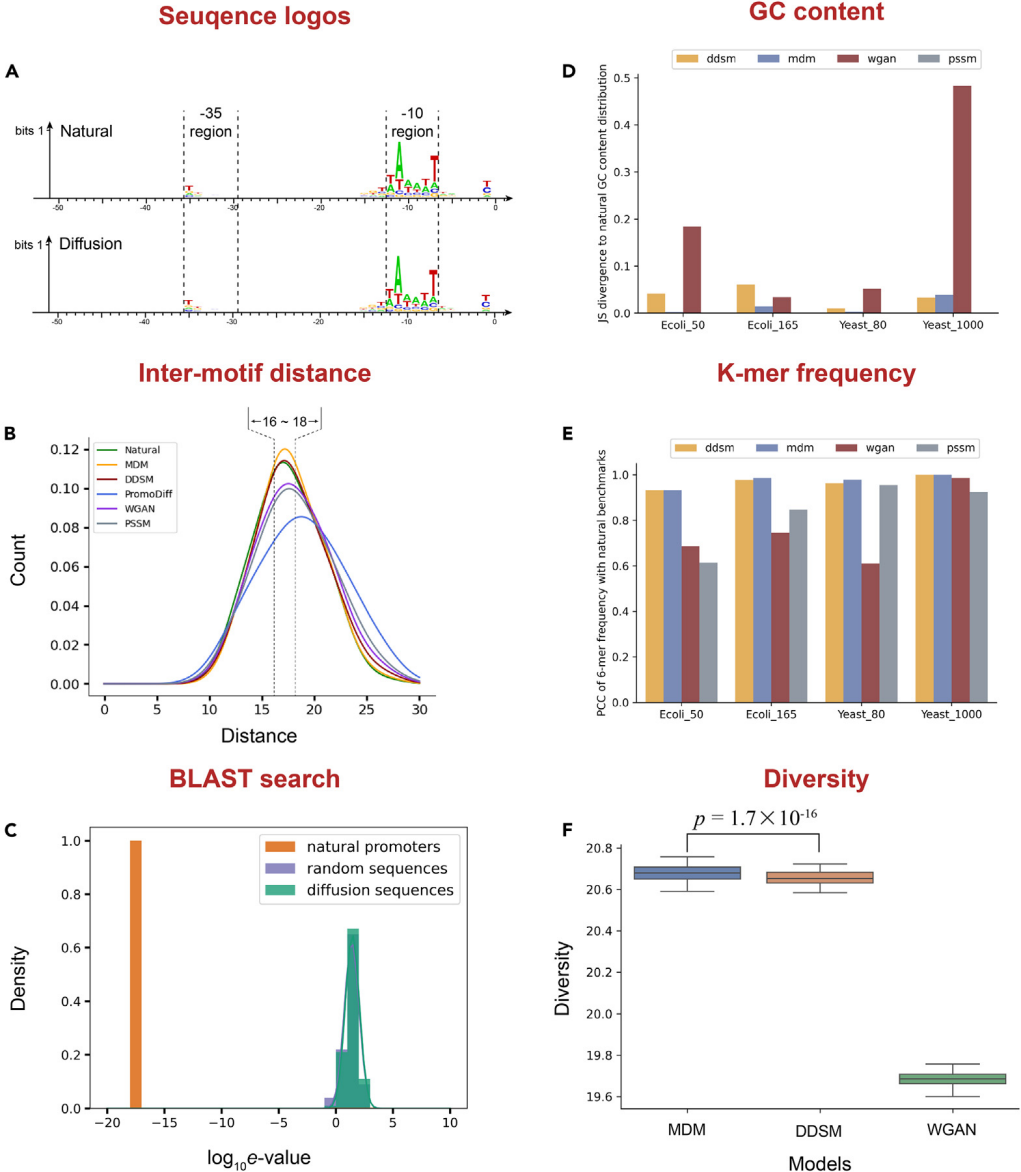
The computational validation of different generative models Subfigures (A–C) represent knowledge-based evaluations, while (D–F) represent data-driven evaluations. A) The sequence logo of natural promoters and MDM-generated artificial promoters. (B) The distribution of inter motif distances between −10 and −35 regions, natural promoters are marked in green, while promoters generated by MDM, DDSM, PromoDiff, WGAN, and PSSM are marked in yellow, red, blue, purple and gray respectively. (C) The results of the BLAST search on natural, MDM-generated and random sequences. (D) The JS divergence of GC content between model-generated promoters and natural, on four benchmark datasets. (E) The Pearson correlation of 6-mer frequency between model-generated promoters and natural. The y axes represent the Pearson correlation value. (F) The average intra-sample distance of samples generated by MDM, DDSM and WGAN, at 100 different random seed. The *p* value represents the significance level under the Wilcoxon signed-rank test.

We firstly evaluated the performance of the MDM under knowledge-based metrics. The core motifs in the ˗10 and ˗35 regions are known to be the most significant features.^29^ By representing the natural and generated promoters with position specific scoring matrix (PSSM) respectively, we found both of them have highly conserve motif features occurring in ˗10 (TATAAT) and ˗35 regions (Figure 2A). Besides, the inter motif distance between the ˗10 and ˗35 regions is typically around 17 bp for better RNA polymerase binding.^29^^,^^30^ We used the MDM, DDSM, WGAN, PSSM, and PromoDiff to generate 10,000 sequences for comparison. The distance distribution of the MDM-generated promoters exhibits the highest enrichment levels around 16–18 bp (Figure 2B), providing a better chance for binding of RNA polymerase. Finally, despite having the ability to mimic biological features of natural promoters, MDM-generated promoters shall be distinct from natural promoters as high level of sequence resemblance will increase the likelihood of homologous recombination. To ensure this global dissimilarity, we performed a standard nucleotide BLAST search, against the *E. coli* K12 MG1655 (taxid:83333). The e-value of random and MDM-generated sequences are at the same level (Figure 2C), indicating that no significant matches occurred.

Then, we benchmarked the MDM’s adaptability on various data-driven metrics. Given that PromoDiff has shown relatively poor results under knowledge-driven metrics, we have chosen WGAN and DDSM for comparison. We evaluated the GC content levels and 6-mer frequencies. For the WGAN model, we selected the last outcome after 12 training epochs, while for the MDM and DDSM, we selected the last outcome after 200 training epochs. In the GC content metrics (Figure 2D), the MDM demonstrates the best performance on the data source with sequence lengths ranging from 50 to 165, but it performed slightly less than the DDSM on the dataset with sequence lengths equal to 1,000. In the 6-mer frequency metrics (Figures 2E and S2), the MDM achieved the best performance across all four benchmarks. These results demonstrate the superiority of the MDM on datasets shorter than the kilo bp level, and we observe that the instability of the WGAN might lead to its unsatisfactory performance. We also employed contrastive learning and t-SNE to embed these outcomes, and MDM-generated promoters exhibited a high level of similarity with natural promoters in feature space (Figure S3). In addition, for practical application, it is desired to obtain highly diverse samples from a single sampling, which facilitates more efficient exploration of the sequence space. In this study, we compared the samples from the MDM, DDSM, and WGAN models under 100 different random seeds, training each model on a 50-bp *E. coli* dataset, with each model generating 10,000 sequences for each seed. We assessed the diversity levels using intra-sample edit distance and created boxplots (Figure 2F). Our results indicate that the MDM model had the highest intra-sample edit distance, suggesting the highest diversity levels.

Overall, MDM has the ability to generate promoters that exhibit important biological features while distinct from natural genomic sequences. Besides, both MDM and DDSM demonstrated superior performance across multiple biological criteria, with MDM performs slightly better than diffusion model based on Dirichlet process, highlighting the superiority of multinomial diffusion architectures in sequence design scenarios. We also found that PromoDiff yields relatively modest results, indicating that directly utilizing discrete diffusion models may be more suitable for DNA sequences, compared to transferring the continuous diffusion models to discrete space. More detailed information about the evaluation can be found in the STAR Methods section.

### MDM ensures robustness in sequence generation

Ensuring the robustness of the sampling process at each training round is crucial for the transition of biological researchers to diverse application scenarios. Here, we trained WGAN, DDSM, and our MDM on the *E. coli* dataset^26^ for 60 epochs, each epoch sample 10,000 sequences, and evaluate the outputs. Here, we trained WGAN, DDSM, and our MDM on the *E. coli* dataset^26^ for 60 epochs. At each epoch, we separately sample 10,000 sequences from each model for evaluation. To quantify the robustness of models, we use the inner edit distance for each sample as a measure of data diversity. In addition, we also assess the model stability by comparing CG content, 6-mer frequency, and poly A/T frequency across outputs of adjacent epochs.

**Figure 3:**
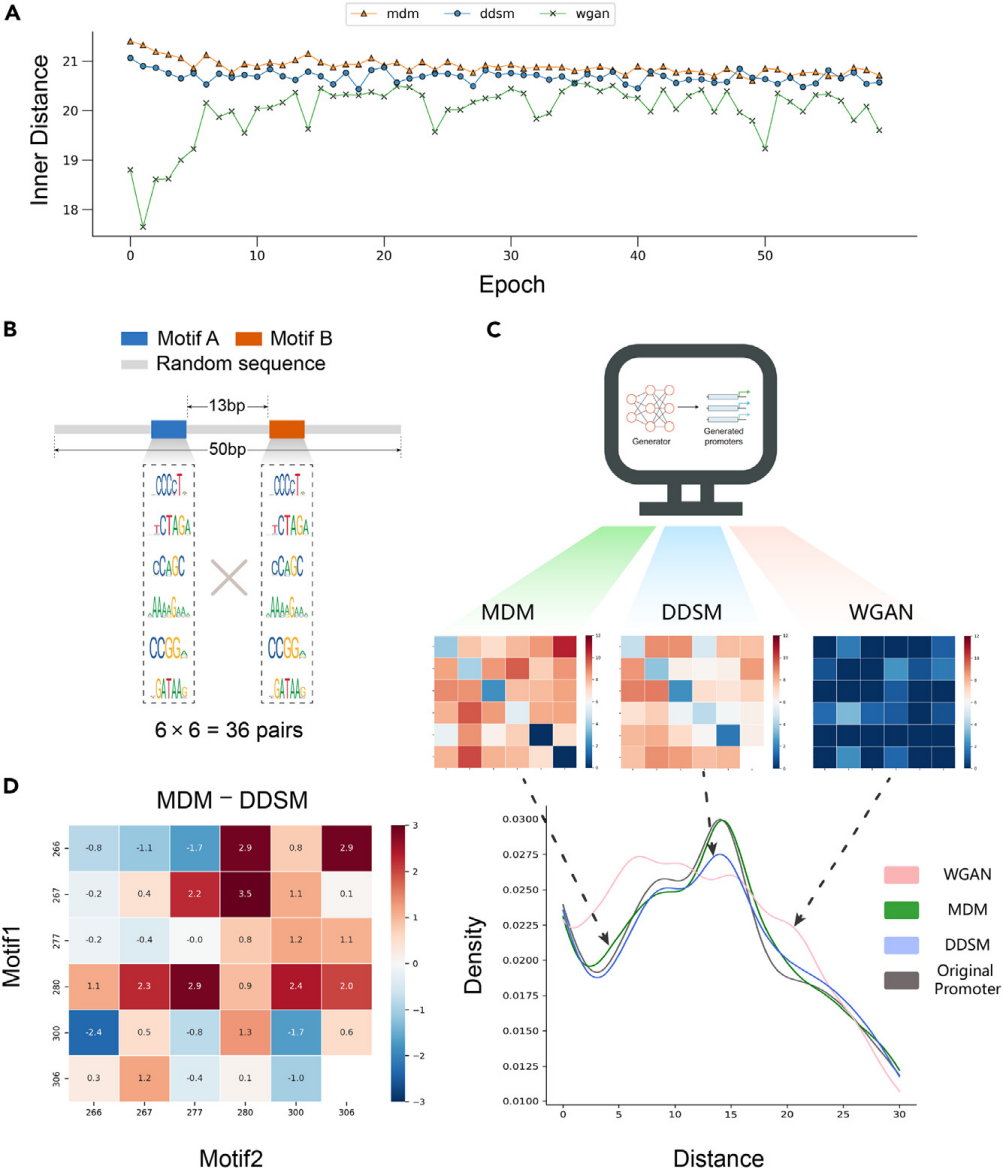
The MDM exhibits robustness and competence in decoupling weak signals (A) The horizontal axis represents the number of epochs trained, the vertical axis represents the intra class diversity of the sampled sequence, the yellow triangle represents the MDM, the blue dot represents the DDSM, and the green cross represents the WGAN. (B) The design of pseudo sequences. (C) The pipeline for comparing different model-generated sequences, each box represents the similarity metric val between the generated and original sequences of a combination. (D) Heatmap of the differences in results between MDM-generated and DDSM-generated sequences, each box represents the $val_{MDM}$ minus $val_{DDSM}$ of a combination. Red dots indicate higher similarity for MDM, while blue dots indicate higher similarity for DDSM.

Our results indicate that both MDM and DDSM outperform WGAN, with MDM demonstrating a slightly higher level of performance (Figure 3A), which is consistent with the aforementioned data-driven analysis. In addition, MDM also maintains a higher level of stability in both CG content and poly A/T frequency between consecutive epochs (Figures S4 and S5). In addition, our results highlight the instability of WGAN. WGAN sometimes even suffers from significant diversity loss, which is strongly attributed to intrinsic mode collapse in GAN models. Mode collapse occurs when the generator and discriminator of a GAN converge to a local minimum,^31^ with the generation of sequences only follow one or a few specific patterns. This phenomenon is particularly severe in discrete data spaces, such as DNA. Previous promoter generation approach with WGAN employed Wasserstein distance, gradient penalty, and residual block to address this issue,^6^ but we still encountered mode collapse during the training process, where every sequence resembled a same pattern like “AAA … AAA”. Here, MDM has converted the estimation process into a transformation from $x_{t}$ to ${\hat{x}}_{0}$, which eliminates the risk of being trapped in local minima.

The aforementioned results indicate that MDM did not experience mode collapse even once across experiments on multiple datasets and various sampling scales. This demonstrates the robustness of MDM, with all its properties offering significant training benefits. Unlike traditional generative models, which often require predetermined experimental training hyperparameters, MDM allows us to set a considerably high number of training epochs without concerning about mode collapse or non-replicable results. This increased robustness provides us with more flexibility and confidence in training the model effectively.

### MDM decouples the weak signal mixing

Decoupling complex motif combinations to decipher hidden biological grammars is currently an essential issue for biological researchers, which also poses challenges for generative AIs. Short motifs in the genome can be perceived as signals, and the functionality of natural DNA is often achieved through the integration of these weak signals. Therefore, the abilities of model to decouple complex signals and preserve natural weak signals are particularly important. We conducted an *in silico* experiment to evaluate these capacities of MDM. Specifically, we utilized 6 motifs from the Fungi genome in JASPAR (STAR Methods) as insertion fragments in our simulation experiments, each motif has a length ranging from 6 bp to 9 bp. We combined these motifs in pairs, resulting in a total of 36 combinations. For each combination, we randomly sampled motif sequences from their position frequency matrices respectively, inserted a 13 bp random sequence between the 2 motifs, and padded at both ends to attain a total length of 50 bp (Figure 3B). In this way, we generated 1,000 distinct sequences for each pair of motifs, and in the end, we obtained a training dataset comprising 36,000 50-bp pseudo sequences with mixed motif features. This simulation dataset served as the training dataset for evaluating the sampling outcomes of MDM, WGAN, and DDSM. We aim to accurately identify all 36 types of signals from the models’ outputs. Here, for each motif pair, if we can detect spacer lengths enriched around 13 bp, this signal will be considered to be captured. Our objective is to ensure that the generated models’ outcomes exhibit a consistent gap distribution with the original dataset, for all 36 pairs of motifs. The training epochs for both MDM and DDSM are set to 100, and are set to 12 for WGAN.

To better visualize the performance, we plotted the experimental results into a heatmap. In the heatmap, value of each element (val) equals the negative logarithm of the cross-entropy (H) between the gap distribution of generated sequences ($dist_{gen}$) and original sequences ($dist_{ori}$):

$$val=-\log_{2}\;H\left(dist_{gen},dist_{ori}\right)$$

Therefore, the redder the color in the heatmap, the higher is the similarity between the generated and original sequences in terms of the gap distribution. Our results suggest that both MDM and DDSM outperforms WGAN (Figure 3C), indicating the superiority of the diffusion model structure over the GAN structure. Besides, MDM outperforms WGAN on 34/36(94.4%) motif pairs and outperforms DDSM on 24/36(66.7%) motif pairs (Figure 3D, also see Figures S6 and S7), suggesting that MDM possesses strong capabilities in capturing and decoupling weak signals. It’s crucial for synthetic biology researchers. MDM prevents the mistaken mapping of high-order grammars into the latent space, which could lead to information loss during reconstruction. This precision is often the key to scientific discoveries in many biological scenarios.

### MDM-generated promoters show high *in vivo* validation rate

**Figure 4:**
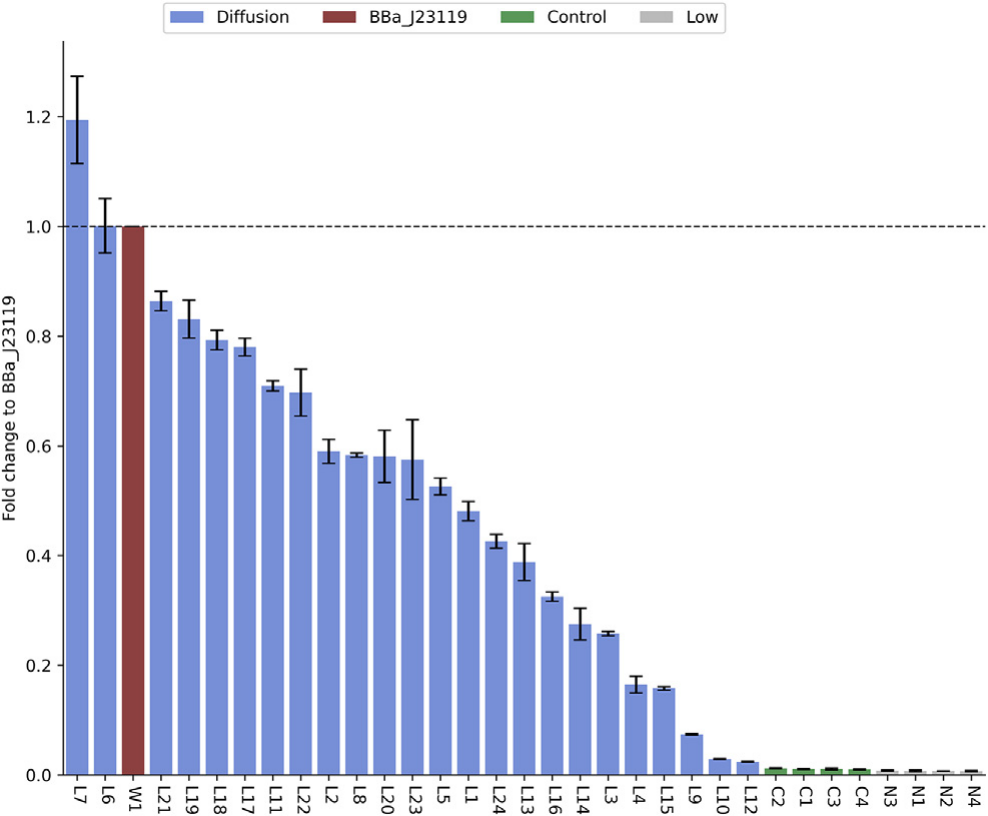
The *in vivo* test of MDM-generated promoters and control promoters MDM-generated promoters are named with prefix L and marked in blue, high-strength wild-type BBa_J23119 is named as W1 and marked in red, completely random sequences with CG content at 50% are named as C1∼C4 and marked in green, sequences with low predicted value by predictor are named as N1∼N4 and marked in gray. Data are represented as mean ± SD from triplicates. All measurements are detailed in Table S1.

We performed an *in vivo* validation on MDM generated promoter in *E. coli* (Figure 4). The 50 bp MDM generated promoter were inserted into a low copy-number plasmid vector containing sfGFP reporter gene and p15A origin of replication. This is to ensure that the expression level of the reporter gene is not masked by the effect of gene copy numbers. A gene insulator RiboJ was inserted within promoter region and ribosomal binding site to ensure that 5′ end of every transcript are identical regardless the nature and effect of synthetic promoter.^32^^,^^33^ Besides, a terminator DT5 was added at the 5′ end of synthetic promoter to prevent the influence of upstream components. The recombinant plasmids were transformed into *E. coli* to perform an *in vivo* study. Parallel to Wang et al., promoter activity can be quantified as the expression level of reporter gene, in this study, it was obtained by measuring the fluorescence intensity of *E. coli* strain carrying targeted promoter.

Twenty four MDM generated promoters that predicted to have high expression level were selected to perform *in vivo* validation and all of them were experimentally demonstrated to be functional. We define functional as having a higher expression level than a 45 bp random DNA sequences that GC content controlled at 50%. Two of the MDM-generated promoters, L7 and L6, showed comparable or even higher promoter activities compared to BBa_J23119, the strongest wild-type constitutive promoter that has been reported in *E. coli*. The highest expression level of MDM-generated promoters achieved 119% activity of BBa_J23119. In addition, several promoters with predicted low expression levels were also selected for *in vivo* validation (N1∼N4). As anticipated, they exhibited low activities similar to random sequences (R1∼R4), proving the efficiency of our predictive model.

Therefore, MDM-generated promoters possess a high validation rate *in vivo*. This demonstrates the effectiveness of our generative AI framework.

## Discussions

In this study, we focused on *de novo E. coli* promoter design with MDM, and provided an organized set of metrics and benchmark datasets for evaluating the performance.

By leveraging advancements in machine learning, we were able to employ a stable generation process that addressed the issue of mode collapse and training difficulties commonly encountered in current approaches. The *in silico* experimental results suggested that MDM effectively explored the functional space of *E. coli* promoters and demonstrated a superior ability to capture significant biological features, to produce novel sequences that differ from natural, to migrate to different biological scenarios, and to produce sequences with a high level of diversity. Besides, MDM could overcome the risk of mode collapse and unstable training process. Additionally, through simulation experiments, we have demonstrated that MDM has the highest competence for deciphering higher-order latent biological signals. Finally, we conducted biological experiments, and results suggested that our MDM-generated sequences exhibited high expression levels and functional probabilities.

The high *in vivo* validation rate demonstrates the effectiveness and potential of MDM. One possible explanation for these promising outcomes is that MDM successfully captured some weak but crucial signals, increasing the likelihood of the generated sequences being functional. Expanding the scale of experiments, seeking the assistance of interpretable methods,^34^ and detecting differential gene expression factors^35^ might be promising ways for further verifying our hypothesis.

In summary, we employed an advanced generative approach MDM for *E. coli* promoter design and conducted evaluations to compare the performance of various deep generative models. Our results suggest that MDM yields promising outcomes in both simulations and biological validations, indicating its potential for further implementations.

### Limitations of the study

This study primarily aims to highlight the promising performance of MDM, as evidenced by computational analysis and *in vivo* validations. However, applying MDM to generate kilobase-level DNA sequences requires considerable computational resources and time. This limitation restricts its application to the *de novo* generation of genome-scale sequences. Moreover, although we benchmarked MDM on biological datasets covering different sequence lengths, regulatory regions, and species, and it generally exhibited superior performance, the key model structures or data features fundamentally affecting its performance across different benchmarks have not yet been identified. These might be barriers to broader implementations of MDM and warrant further discussions and investigations.

## Resource availability

### Lead contact

Further information and requests for resources and reagents should be directed to and will be fulfilled by the lead contact, Xiaowo Wang (xwwang@tsinghua.edu.cn).

### Materials availability

• Plasmids generated in this study have been deposited to Benchling website:https://benchling.com/poonmaynee/f_/MCwerMW9-synthetic-promoter-design-in-escherichia-coli-based-on-multinomial-diffusion-model/.
• This study did not generate new unique reagents.

### Data and code availability

• Benchmark datasets needed to replicate simulations have been preprocessed and uploaded to GitHub and Zenodo. DOIs are listed in the key resources table. The original dataset sources for benchmarks have been made accessible by their original research group. The accession numbers of these datasets are listed in the key resources table. The raw data for robustness analysis, weak signal decoupling, and samples for biological validation in this study cannot be deposited in a public repository due to its large size. Instead, we have provided the relevant code for reproduction. Processed results for biological validation are provided in supplementary materials.
• The original code for computational validations has been deposited on GitHub and is publicly available as of the date of publication. The relevant toolkit, GPro, for efficiently reproducing generative and predictive models has been publicly available, complete with a wiki as a detailed tutorial.
• Any additional information required to reanalyze the data reported in this paper is available from the lead contact upon request.

## Acknowledgments

This work was supported by the 10.13039/501100001809National Natural Science Foundation of China (no. 62250007, 62225307,and 61721003), and the grant from the 10.13039/100020721Guoqiang Institute, Tsinghua University.

## Author contributions

Q.X.D. and X.W.W. conceptualized the research. Q.X.D. performed major computational validations. M.N.P conducted the *in vivo* biological validations. Q.X.D. and M.N.P. wrote and revised the manuscript. X.C.Z. plotted the main figures, H.C.W. and Y.W. assisted in organizing the computational evaluation approaches, P.C.Z. and Y.W. provided guidance for *in vivo* validations, and Z.W. and L.W. helped with the manuscript revisions.

## Declaration of interests

All authors declare no competing interests.

## STAR★Methods

### Key resources table

**Table:** 

REAGENT or RESOURCE	SOURCE	IDENTIFIER
**Bacterial and virus strains**		

NEB® 5-alpha Competent *E. coli* (High Efficiency)	New England Biolabs	Cat#C2987H

**Chemicals, peptides, and recombinant proteins**		

Ultra™ II Q5® Master Mix	New England Biolabs	Cat#M0544S
NEBuilder® HiFi DNA Assembly Master Mix	New England Biolabs	Cat#E2621S
Adilab High Pure Plasmid Mini Kit	Adilab	Cat#PL03
Adilab Gel Extraction Kit	Adilab	Cat#DR01

**Deposited data**		

Processed benchmarks	This study	https://doi.org/10.5281/zenodo.13847934
Raw data for *Escherichia coli* K12 MG1655 (50 bp promoters)	Thomason et al.^18^	https://doi.org/10.1128/JB.02096-14
Raw data on *Escherichia coli* K12 MG1655 (165 bp promoters)	Johns et al.^19^	https://doi.org/10.1038/nmeth.4633
Raw data on yeast *Saccharomyces cerevisiae* (80 bp core promoters)	Vaishnav et al.^5^	https://doi.org/10.1038/s41586-022-04506-6
Raw data on yeast *Saccharomyces cerevisiae* (total 1000 bp regulatory sequences)	Zrimec et al.^9^	https://doi.org/10.1038/s41467-022-32818-8
Information needed for replicating computational evaluations (e.g., motifs from JASPAR)	This study	https://doi.org/10.5281/zenodo.13847934

**Oligonucleotides**		

Primers for *in vivo* validation, see Table S2	This paper	N/A

**Recombinant DNA**		

Plasmid: pMDM-P15A-DT5-RiboJ-B0034-SFGFP-B0015	This paper	https://benchling.com/poonmaynee/f_/MCwerMW9-synthetic-promoter-design-in-escherichia-coli-based-on-multinomial-diffusion-model/

**Software and algorithms**		

Multinomial diffusion model (MDM, including all codes needed for replication)	This study	https://github.com/WangLabTHU/MDM
GPro Python package (Including models for MDM, WGAN and predictor)	Wang et al.^20^	https://github.com/WangLabTHU/GPro
Dirichlet diffusion sore model (DDSM)	Avdeyev et al.^13^	https://github.com/jzhoulab/ddsm
Wasserstein generative adversarial network (WGAN)	Wang et al.^8^	https://github.com/WangLabTHU/Deep_promoter
PromoDiff	Wang et al.^14^	https://github.com/wangxinglong1990/Promoter_design/
Nucleotide BLAST	National Library of Medicine	https://blast.ncbi.nlm.nih.gov/Blast.cgi
WebLogo 3	University of California, Berkeley	https://weblogo.berkeley.edu/logo.cgi
Pytorch Python package	PyTorch Foundation	https://pytorch.org/
Anaconda 3	Anaconda, Inc.	https://www.anaconda.com/
Snapgene	Dotmatics	https://www.snapgene.com/

**Other**		

Varioskan FLASH	Thermo Scientific	Cat#N06354

### Method details

#### MDM training process

Multinomial diffusion model is a type of generative model that reconstructs samples from completely random initial sequence.^22^ In this model, at each time step t, a vector $x_{t}$ is represented in a one-hot encoded format, where $x_{t}\in {\left\{0,1\right\}}^{K}$. Specifically, for category k, $x_{k}=1$, and $x_{j}=0$ for j≠k.

This representation allows the diffusion process to be applied to discrete spaces. The process of adding noise in multinomial diffusion can be described as a state transition equation, where the current state $x_{t}$ is obtained by sampling from the previous state $x_{t-1}$ by adding noise:

$$q\left(x_{t}|x_{t-1}\right)=C\left(x_{t}|\left(1-\beta_{t}\right)x_{t-1}+\frac{\beta_{t}}{K}\right)$$

The parameter $\beta_{t}$ determines the probability of uniformly resampling a category. K is the number of categories, which in this case is 4 bases for DNA (A, T, C, G), and C denotes a conditional categorical distribution with defined parameters.

Since these noise processes follow a Markov chain, the conditional distribution of $x_{t}$ can be defined by $x_{0}$, where $\alpha_{t}=1-\beta_{t}$, and ${\bar{\alpha }}_{t}=\prod_{\tau =1}^{t}\alpha_{\tau }$:

$$q\left(x_{t}|x_{t-1}\right)=C\left(x_{t}|{\bar{\alpha }}_{t}x_{0}+\frac{1-{\bar{\alpha }}_{t}}{K}\right)$$

Hoogeboom et al.^22^ provided the format of the categorical posterior distribution $q\left(x_{t-1}|x_{t},x_{0}\right)$. During the reverse denoising process, the parameters in the generative model can be estimated by minimizing the discrepancy between $p_{\theta }\left(x_{t-1}|x_{t}\right)$ and $q\left(x_{t-1}|x_{t},x_{0}\right)$^36^. The loss function of diffusion model can be computed through variational inference:

$$\log\;p_{\theta }\left(x_{0}\right)\geq E_{q}\left\{\log\;p_{\theta }\left(x_{0}|x_{1}\right)-KL\left[q\left(x_{T}|x_{0}\right)\|p_{\theta }\left(x_{T}\right)\right]-\sum_{t=2}^{T}KL\left[q\left(x_{t-1}|x_{t},x_{0}\right)\|p_{\theta }\left(x_{t-1}|x_{t}\right)\right]\right\}$$

Since the trajectory q is well defined, the Kullback-Leibler divergence (KL divergence) between $q\left(x_{T}|x_{0}\right)$ and $p_{\theta }\left(x_{T}\right)$ is approximately zero.^22^ Rather than directly training θ, we employed transformer-based models to predict ${\hat{x}}_{0}$ from ${\hat{x}}_{t}$ and timestep t, through ${\hat{x}}_{0}=\mu \left(x_{t},t\right)$. This prediction is used to parameterize $p_{\theta }\left(x_{t-1}|x_{t}\right)$. Therefore, the optimization can be achieved through minimizing the KL divergence for discrete distributions in the whole diffusion trajectory. Here we used L to symbol the total loss, and $L_{t-1}$ to symbol the KL loss at denoising step for $x_{t-1}$:

$$L=E_{q}\left\{-\log\;p_{\theta }\left(x_{0}|x_{1}\right)+\sum_{t=2}^{T}KL\left[q\left(x_{t-1}|x_{t},x_{0}\right)\|p_{\theta }\left(x_{t-1}|x_{t}\right)\right]\right\}=\sum_{t=1}^{T}L_{t-1}$$

$$L_{t-1}=KL[q\left(x_{t-1}|x_{t},x_{0}\right)||p_{\theta }\left(x_{t-1}|x_{t}\right)]=KL[q\left(x_{t-1}|x_{t},x_{0}\right)\|q\left(x_{t-1}|x_{t},{\hat{x}}_{0}\right)],t=1,2,\ldots ,T-1$$

We noted that this format also works for $L_{0}$, since the categorical posterior can be computed in closed-form^22^:

$$L_{0}=-\log\;p_{\theta }\left(x_{0}|x_{1}\right)=-\sum_{K}x_{0,k}\;\log\;{\hat{x}}_{0,k}=\sum_{K}x_{0,k}\left[\log\;x_{0,k}-\log\;{\hat{x}}_{0,k}\right]=KL\left[C\left(x_{0}\right)\|{\hat{x}}_{0}\right]$$

Then after the computation of loss item $L_{t}$, the loss function can be minimized by training a well-performed MDM. For model training, we tokenized $x_{0}$ using one-hot encoding and then applied positional embedding, yielding the result $m\left(x_{0}\right)$. Furthermore, we utilized positional embedding for all time steps t, ultimately obtaining the embedding of n(t). Here $h_{t-1}$ is used to represent the embedding at timestep t−1, and ψ to symbol all the model blocks, yielding the following equation:

$$h_{t}=\psi \left(h_{t-1}+n\left(t\right)\right),h_{0}=m\left(x_{0}\right),t=1,2,3,\ldots ,T$$

Here, the default batch size is set to 64, the learning rate to 1e-4. We employ a 12-layer transformer with 16 heads per layer. The diffusion step is set to 100, and the local size for the transformer is configured to be divisible by the sequence length (e.g., 25 for 50 bp E. coli dataset). A batch of N input DNA sequences, each with a sequence length of L, will be transformed into one-hot encoding, resulting in a tensor with dimensions (N×L×4). The default number of training epochs in this paper is 100. By default, the MDM automatically selects 90% of the total sequences for training, and reserves the remaining 10% for evaluation (saved in logs) during the training step. Specifically, we use the GPro package^28^ to efficiently replicate this model. A few-lines Python code for implementing training on 50 bp E. coli benchmark can be simplified as follows:

mdm = Diffusion_language(length = 50, transformer_local_size = 25, epochs = 100, model_name = "mdm", batch_size = 64).

mdm.train(dataset = dataset_path, savepath = checkpoint_path).

After training process, for generation, we randomly generated multiple sequences as ${\hat{x}}_{T}$ based on 0-order Markov chain. The denoised sequences ${\hat{x}}_{0}$ are the required novel samples. All aforementioned presents the primary framework employed by MDM, and the model’s architecture is illustrated in Figure 1. After training with the GPro implementation, a one-line Python code can be used to generate sequences:

mdm.generate(sample_model_path, sample_number = 10000, seed = 0).

In general, the potential sequence length generated by MDM can reach thousands of base pairs, but due to the complexity of model architecture and CUDA memory constraints, we typically recommend generating sequences for a few hundred base pairs. The major computational bottleneck lies in the sampling process rather than in training. On a single computational node with a GeForce RTX 3090, the maximum sampling batch for the 1,000 bp yeast benchmark is 8, whereas for a 50 bp sequence, the sampling batch is 10,000. This indicates an exponential growth in computational resource requirements as the sequence length increases. To assess these performance limits, we conducted more detailed simulations on all benchmarks discussed in this paper. These performance analyses have been published in the wiki of our toolkit GPro.^28^.

#### Predictor training process

The predictive model primarily aligns with that of Wang et al.,^6^ with a slight improvements in model architecture but the same Pearson correlation value on the 50 bp E. coli benchmark. The predictive model is implemented using the Python package PyTorch, with four 1D-convolutional neural network architectures. Each 1D-convolutional layer has the same kernel size of 5, stride of 1, padding size of 2, and the output channels are 100, 200, 200, and 10, respectively. After each convolutional layer, a batch normalization layer was employed, followed by a rectified linear unit (RELU) nonlinearity. The outputs are finally down-sampled using a max-pooling layer, flattened, and then passed through a linear fully connected neural network to obtain a single value output. The default batch size is set to 64, training epoch is 200, and the learning rate to 1e-5, and training results are saved at every 20 epochs. For predictive model training, the *E. coli* 50 bp benchmark is deduplication to 11,000 sequences, subsequently be partitioned into subsets with a ratio of 0.56:0.24:0.2. We also set an early stopping criterion of 50 epochs to ensure that if the model does not improve its Pearson correlation in the long term, it will stop training to save time. We have also encapsulated this model in GPro for efficient replication, a few-lines Python code for training and predicting can be simplified as follows:

cnn_k15 = CNN_K15_language(length = 50, epoch = 200, patience = 50).

cnn_k15.train(dataset = dataset, labels = labels, savepath = save_path).

res = cnn_k15.predict_input(model_path = model_path, inputs = data_path).

This model is particularly suitable for short sequences (50, 80, 165 bp) and exhibits excellent performance. We have also compared its performances with other previously proposed predictive models on all four benchmarks employed in this paper, and these comparisons have been published in the wiki of our toolkit, GPro.^28^

#### Analysis of sequence logos

Sequence logos are graphical representations of multiple DNA/protein sequence alignments, with one stack for each position in the sequence. The height of the symbols at each position represents the relative frequency of each amino acid or nucleotide, and a region where each position has a dominant amino acid or nucleotide represents a conserved region. Crooks et al.^37^ from Berkeley provided the WebLogo3 webpage for efficiently generating sequence logos. We uploaded 10,000 sequences for both the natural training dataset (50 bp *E. coli* benchmark) and the MDM-generated sequences (diffusion) separately, and set the logo range from −50 to −1, with the first position number set to −50. The highly conserved regions in both the −35 and −10 regions of natural sequences are precisely captured, while the occurrence of other conserved positions is also efficiently captured in the region near the TSS.

#### Analysis of inter-motif distance

Two significant functional motifs in the −10 and −35 regions upstream of the TSS in *E. coli* are among the most significant features for the binding of RNA polymerase. The inter-motif distance between these regions should typically be around 17 bp for optimal biological function. Firstly, we utilized pretrained generative models (MDM, DDSM, WGAN, PromoDiff) with the training settings provided in the main article to generate 10,000 sequences. We used the total 14,098 sequences from the 50 bp *E. coli* benchmark as the natural wildtype and calculated its position-specific scoring matrix to generate an additional 14,098 sequences for comparison. The original positional frequency matrices (PFM) of −10 and −35 motifs are from Wang et al.,^6^ and we employed Python package Bio to quantify the occurrence of specific motifs in any region. The background distribution of each base is set as "T":0.291, "C":0.218, "G":0.225, "A":0.266, while the threshold for defining the occurrence is set to −1,000 to avoid false negatives. For each sequence, we used a Gaussian distribution kernel from Python package scipy to fit a smooth density function to the inter-motif distances, setting the covariance factor to 0.5. The outputs of MDM show the highest enrichments in these regions, while those of PromoDiff show the poorest.

#### Analysis of BLAST search outcomes

We measure the similarity levels of *de novo* designed sequences to the natural *E. coli* genome using the basic local alignment search tool (BLAST) webpage. Specifically, we selected the standard nucleotide BLAST program, uploaded our designed sequences, and searched the “nucleotide collection” database for the organism *Escherichia coli* MG1655 (taxid:511145). We set the search program to “somewhat similar sequences” (blastn) and the expect threshold to 1,000. Since the *de novo* designed sequences are highly dissimilar to natural ones, a small threshold might lead to no results. We uploaded 10,000 sequences from the *E. coli* benchmark, MDM outputs, and random sequences, and plotted the distribution of all the E-values. The *de novo* designed sequences are highly dissimilar to natural sequences, indicating a low risk of false homologous recombination.

#### Comparisons of GC content

The maintenance of GC content (the occurrence of nucleotide G and C) is significant for downstream analysis, as too high or too low GC content can harm the assembly, gel electrophoresis, and PCR processes. To quantify the GC content between model-generated sequences and training inputs, we evaluated the sampling of 10,000 sequences from MDM, DDSM, and WGAN on all four benchmarks. For each sampling, firstly we randomly sampled sequences from the benchmark for comparison at three random seeds, with the number of comparison sequences set to the minimum of the benchmark and sampling scales. We then quantify the percentage of GC content for each sequence, generating lists of GC contents for both natural and model-designed sequences. Subsequently, we quantify the Jensen–Shannon divergences (JS divergences) between these two lists and, finally, obtain an averaged JS divergence across the total of three replications, which uses the form:

$$\text{js}\_\mathrm{div}\left(i\right)=\frac{1}{2}KL\left(P_{i}\|M_{i}\right)+\frac{1}{2}KL\left(Q_{i}\|M_{i}\right)$$

$$\text{avg}\_\text{js}=\frac{1}{K}\sum_{i=1}^{K}\text{js}\_\mathrm{div}\left(i\right)$$

Here $P_{i}$ and $Q_{i}$ represent the random selections of n=min(len(P),len(Q)) samples from GC content lists P and Q, respectively; $M_{i}$ represents the average of $P_{i}$ and $Q_{i}$, and K=3 indicates the totaling 3 different random seeds. The calculation of KL divergence is through Python package scipy. Our results demonstrate that MDM outperforms all other models in short sequences, with only a slight performance loss at the kb level.

#### Comparisons of k-mer frequency

Ensuring that k-mer fragments (all possible fragments with a length of k) in natural sequences have the same occurrence opportunity in model-designed sequences is crucial for maintaining the biological grammars. We measure this maintenance ability by comparing the Pearson correlation coefficient between two lists of k-mer frequencies from natural and model-designed sequences. For all benchmarks, we employ MDM, DDSM, and WGAN to sample 10,000 sequences for evaluation, with PSSM as the negative control. We quantify the 6-mer frequency list for each sampling, and compare these lists with the natural frequency list to calculate the Pearson correlation coefficient. We visualize the results with both a scatterplot with a regression line (supplementary) and a bar plot. The results suggest that although PSSM sampling can maintain a high level of GC content, it lacks the ability to capture significant fragments. Both diffusion models outperform the WGAN model, while MDM demonstrates higher performance across benchmarks of all sequence lengths.

#### Comparisons of data diversity

Sampling with high data diversity enables more efficient exploration of the biological landscape and helps to select a smaller pool of promising candidates for validation, thus saving time and expense. In this study, we leverage the Python package Levenshtein to quantify the edit distance between each sequence within the sampling set, as a measurement of sequence diversity. Specifically, we firstly trained the MDM, DDSM, and WGAN models on the 50 bp *E. coli* benchmark, with training durations of 100, 100, and 12 epochs, respectively. For each model, we generated 10,000 sequences for each of the 100 distinct random seeds. For each sampling, we compute the minimum edit distance between each sequence and every other sequence in the set. We thus compile a list of these distances and compute the average, which serves as a measure of diversity for that particular sampling. For instance, when performing MDM sampling with a sampling scale of *N* = 10,000 and a random seed of k, the diversity can be calculated as follows:

$$\min\_\text{dist}\left(i\right)=\underset{j\neq i}{\min\;}\text{Levenshtein}\left(seq_{i},seq_{j}\right)$$

$$\text{avg}\_\text{dis}\left(\text{MDM},\text{seed}=k\right)=\frac{1}{N}\sum_{i=1}^{N}\min\_\text{dist}\left(i\right)$$

Finally, we create an average distance list for each of the three models, with each list containing 100 items. We then boxplot the results for visualization. The results highlight the sampling diversity of diffusion models, with MDM exhibiting a higher diversity in sampling. The *p*-value represents the superiority of MDM’s diversity over DDSM under the Wilcoxon signed-rank test, performed by Python package scipy.

#### Simulations for robustness evaluation

Samples from the outputs of each epoch in a specified training process are used to measure the robustness and stability of the model’s parameters. We trained MDM, DDSM, and WGAN for 60 epochs each on the 50 bp *E. coli* benchmark separately, collecting a total of three groups of samples. For each sample at epoch t, we measured: (1) the inner edit distance, (2) the level of GC content, and (3) The occurrence of poly-A/T fragments, which indicates the presence of four or more consecutive nucleotides A or T, might pose risks for sequence synthesis or downstream assembly. We computed the metrics and graphed their trends. MDM showed the most consistent and highest diversity, with stable GC content and poly-A/T metrics.

#### Simulations for decoupling mixed weak signals

We retrieved six motifs, spanning lengths of 6–9 nucleotides, from the Fungi genome using the JASPAR database for simulation purposes. The motifs included are MA0266, MA0267, MA0277, MA0280, MA0300, and MA0306. We obtained the most recent PFM files for these motifs. All motifs are paired, resulting in a total of 36 unique motif pairs. For a given motif pair (x, y), the procedure for designing simulation sequences is as follows: (1) finalize fragments X and Y by sampling from the positional frequency matrices x and y, (2) randomly generate a 13 bp sequence G, (3) randomly generate a 50 bp template H, and randomly select an equal-length region to replace with X + G + Y. (4) repeat step (1)-(3) for 1,000 times. We generated a simulation dataset of 36,000 50-bp pseudo sequences, which were then trained using MDM, WGAN, and DDSM with 100, 100, and 12 epochs, respectively. For each model, we sampled a batch of 36,000 sequences for comparison. Ideally, the sample should also include a mixture of all 36 motif pairs, with each represented by 1,000 sequences. We quantify the occurrence of each motif pair (x, y) in each sequence using the Python package Bio, and we use the entropy between the length distribution of fragment G model-generated sequences and ($dist_{gen}$) original sequences ($dist_{ori}$) as a measure of the maintenance of the specific grammar (x, y). The results are depicted in heatmaps using the Python package Seaborn, with the color map set to RdBu_r. The minimum value is set to 5.5, and the maximum value is set to 18.

#### Construction of recombinant plasmid

We designed a pair of oligo primers, the forward primer contains 40 nt of the MDM generated promoter from its 3′ end and a 20 nt homologous overlapping region with the vector (C1: 5′-AGCTGTCACCGGATGTGCTT-3′), the reverse primers contain 40 nt from the 3′ end of the promoter’s complementary strand and a 26 nt homologous overlapping region (C2: 5′-GGACATGCGATCGTATTGCCTATGAG-3′), detailed primer sequences for all sample were listed in T. The plasmid vector was linearized with the aforementioned primer pairs through standard endpoint PCR (NEB Ultra II Q5 Master Mix, Cat#M0544S, $T_{m}$ = 70 °C), generating a linear strand with a 30 bp of overlapping area at both ends. The linear strand was then circularized via Gibson assembly (NEBuilder HiFi DNA Assembly Master Mix, Cat#E2621S). The products from Gibson assembly were transform into NEB 5-alpha Competent *E. coli* (Cat#C2987H). Transformant were streaked on chloramphenicol plate for monoclonal selection and inoculate into Luria-Bertani medium with 50 ug/mL chloramphenicol and shake overnight. Plasmid were extracted using commercial kit (Adilab High Pure Plasmid Mini Kit, Cat#PL03) and subjected to Sanger sequencing (genewiz) using both forward (S1: 5′-cgcccggtagtgatcttatt -3′) and reverse (SR: 5′-gacttgaagaagtcatgctg-3′) sequencing primers for verification. Bacteria strains with correct insert were stored as glycerol stocks.

#### Sample preparation

Bacteria strains were picked from glycerol stock using a sterilize tip and inoculate into 5 mL Luria-Bertani medium. Overnight liquid cultures were diluted with a ratio of 1:100 and shake for 8 h, in this step, three biological replicates were prepared. 200 μl of liquid culture sample were added into each well of 96-well flat clear bottom microplate (Corning, Cat#3603).

#### Intensity measurement

A spectral scanning multimode reader (Thermo scientific Varioskan Flash, Cat#N06354) was used to measure the fluorescence intensity and optical density at 600 nm of bacteria sample. Fluorescence intensity of reporter gene sfGFP was measured at an excitation wavelength of 485 nm and emission wavelength of 510 nm. Relative promoter activities were calculated as:

$$S=\frac{{\left(F/OD\right)}_{clone}-{\left(F/OD\right)}_{blank}\;}{{\left(F/OD\right)}_{BBa\_J23119}-{\left(F/OD\right)}_{blank}}$$

The results of the calculations are provided in Table S1.

### Quantification and statistical analysis

#### Statistical analyses

All statistical analyses are mainly performed by Python package scipy, including JS divergence of GC contents (scipy.stats.entropy), the Pearson correlation of 6-mer frequency (scipy.stats.pearsonr), the sequence diversity in model samplings (scipy.stats.wilcoxon), entropies in weak signal decoupling (scipy.stats.Gaussian_kde), etc. Information related to specific statistical tests is listed in the figure legends and/or method details. Data are represented as mean ± SD in bar plots, as indicated in the figure legends. The *p*-values related to Wilcoxon signed-rank test are listed on figures.

#### GPro package for efficient replications

GPro is a user-friendly Python package that systematically encapsulates a collection of cutting-edge, GenAI-empowered models for promoter design. This toolkit offers a standardized pipeline that covers the essential processes of promoter design, including training, optimization, and evaluation. GPro enables the replication of MDM, WGAN, predictors, evaluates k-mer frequency, and provides all benchmark datasets along with performance comparisons of various models. The relevant codes and DOI are listed in the key resources table.

#### Compute environment

All codes are written in Python, using Anaconda as the environment manager. Virtual environments can be directly configured by installing the GPro package. The environment installation archive for offline machine access has been uploaded to Google Drive, and detailed guidance is provided in the wiki. All these resources are provided in key resources table.

#### Computational hardware information

All computations are performed on NVIDIA Geforce RTX 3090 GPU. Detailed information about settings of hardware is available upon request.

## Supplemental information

Document S1. Figures S1–S7

Table S1. Fluorescence intensity measurement, related to STAR Methods

Table S2. Primer sequences, related to STAR Methods
