# Supplementary material for: Synthetic promoter design in Escherichia coli based on multinomial diffusion model
Source: iScience. 2024 Oct 18;27(11):111207. doi: 10.1016/j.isci.2024.111207 (PMC11550136; doi:10.1016/j.isci.2024.111207)
Supplement: Document S1. Figures S1–S7 [file mmc1.pdf]

iScience, Volume *27*

Supplemental information

Synthetic promoter design in *Escherichia* *coli* based on multinomial diffusion model

Qixiu Du, May Nee Poon, Xiaocheng Zeng, Pengcheng Zhang, Zheng Wei, Haochen Wang, Ye Wang, Lei Wei, and Xiaowo Wang

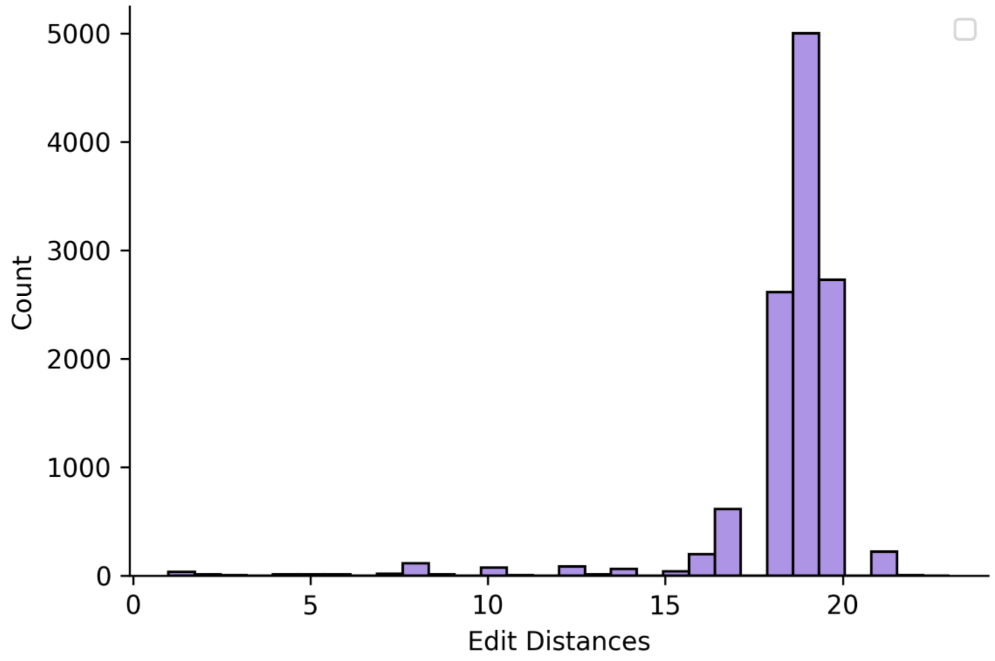

Figure S1. The inner edit distance within E. coli 50 bp benchmark. The calculation of internal sequence similarity employs the “minimum edit distance metric” as described in the STAR Methods. The majority of natural sequences maintain a distinct level with at least a 15-bp difference out of the total 50 bp, thereby reducing the potential for strong bias in the model caused by similar training data.

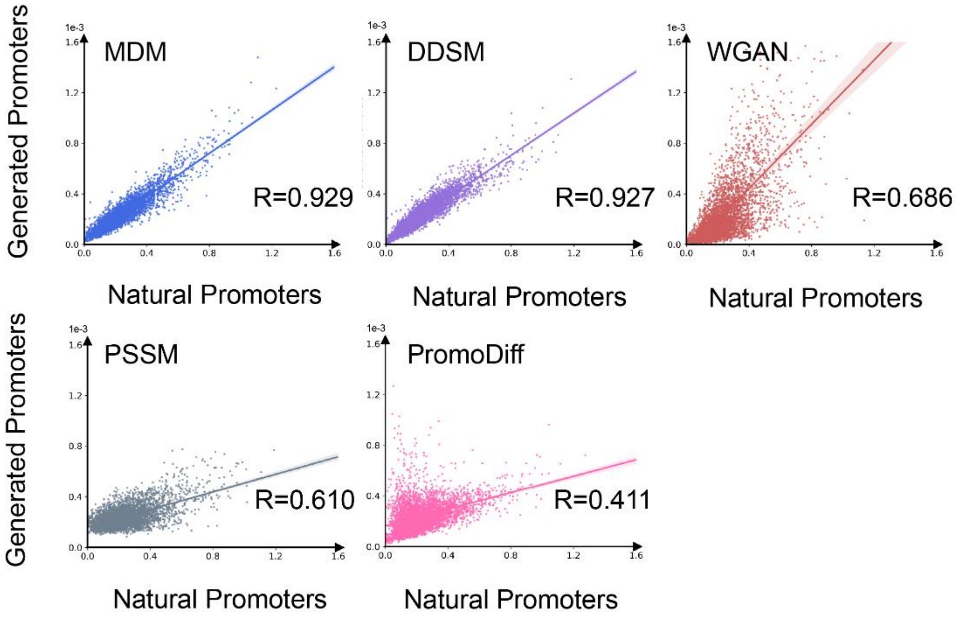

Figure S2. Regression analysis of 6-mer frequency between model-generated promoters and the E. coli 50 bp benchmark, related to Figure 2. The R value represents the Pearson correlation coefficient. Models MDM, DDSM, and WGAN were trained on the E. coli 50 bp benchmark for 100, 100, and 12 epochs, respectively. PromoDiff was initialized with the authors' pre-trained checkpoints. PSSM was directly sampled from the benchmark's positional frequency matrix. MDM and DDSM exhibited the top performance, followed by WGAN, with PromoDiff showing the poorest results. We will not further discuss the PromoDiff model in subsequent sections.

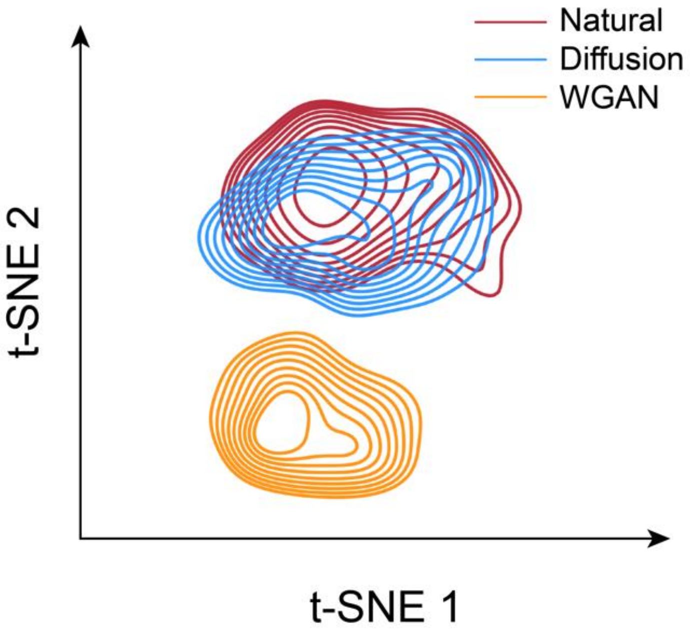

Figure S3. Promoters in the low dimensional embedding space, related to Figure 2. We randomly chose 10,000 natural, MDM-generated, and WGAN-generated promoters, represented these sequences using contrastive learning, and then embedded them into a two-dimensional latent distribution with t-SNE. In the embedding space, red contours represent natural promoters, blue contours represent promoters generated by MDM, and yellow contours represent promoters generated by WGAN. The significant similarity between the embeddings of MDM-generated and natural promoters further demonstrates our model's capacity in capturing the complex features of natural promoters.

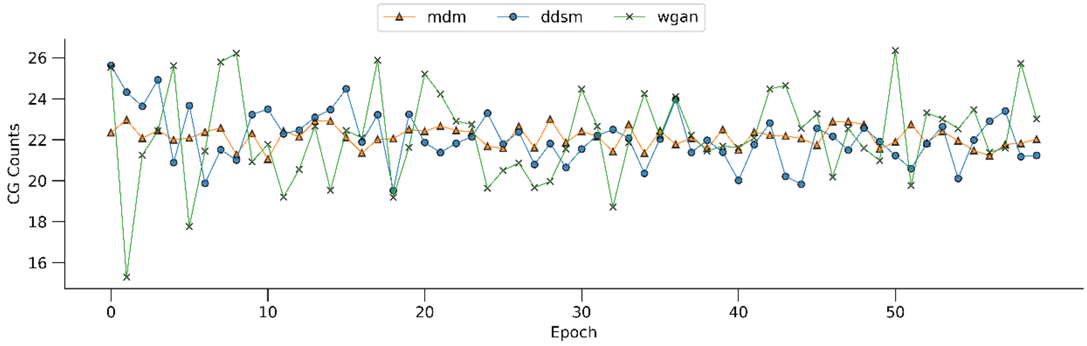

Figure S4. The variation in CG content (see STAR Methods) from the sampling of 60 training epochs of MDM, DDSM, and WGAN, all trained on the E. coli 50 bp benchmark, related to Figure 3. Each sample consists of 10,000 sequences. The yellow triangle represents the MDM, the blue dot represents the DDSM, and the green cross represents the WGAN.

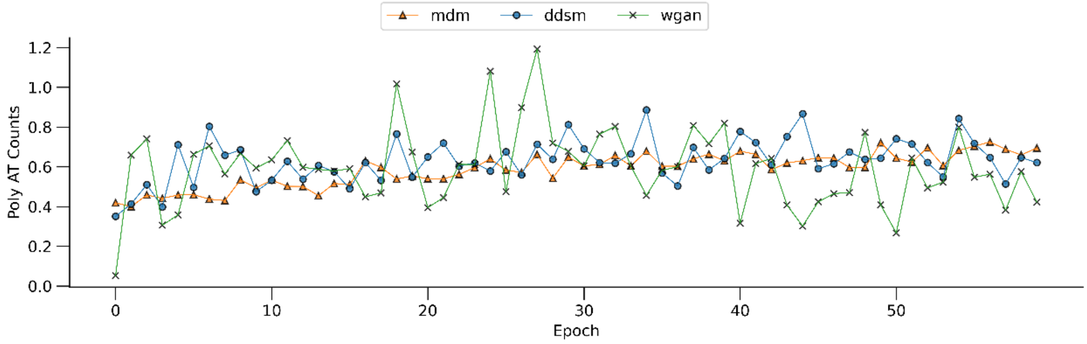

Figure S5. The variation in poly A/T fragment numbers (see STAR Methods) from the sampling of 60 training epochs of MDM, DDSM, and WGAN, all trained on the E. coli 50 bp benchmark, related to Figure 3. Each sample consists of 10,000 sequences. The yellow triangle represents the MDM, the blue dot represents the DDSM, and the green cross represents the WGAN.

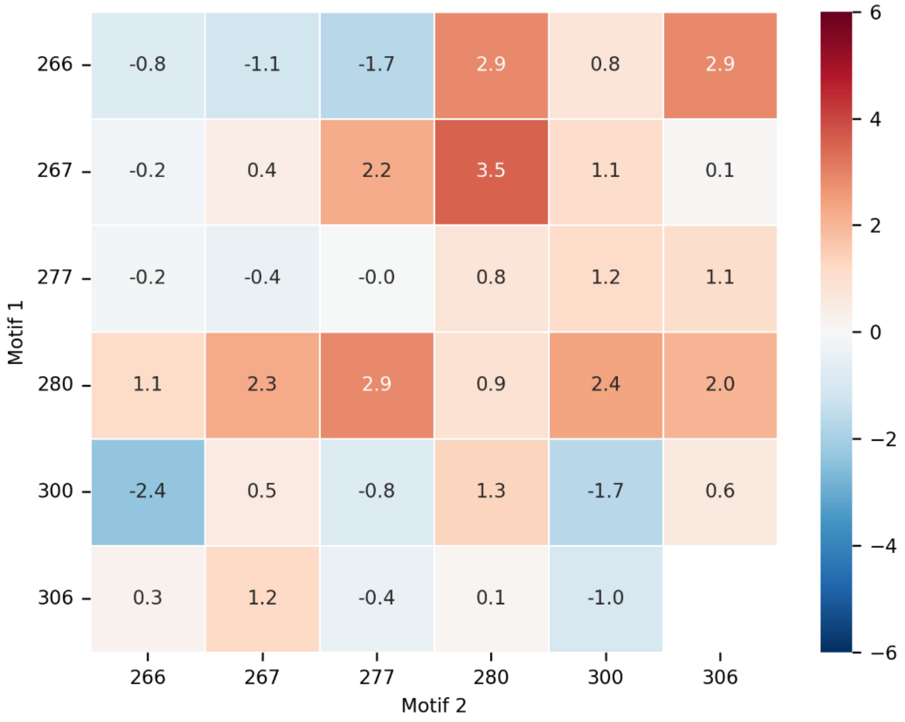

Figure S6. The detailed heatmap of the differences in entropy values between MDM and DDSM (as described in the main text), providing precise float values and detailed motif labels. The heatmap uses the RdBu_r color scheme, with redder shades indicating higher similarity between generated and original sequences in specific combination. In addition, the DDSM model did not generate any effective data for the 306-306 combination, resulting in a NaN value and the blank area in heatmap.

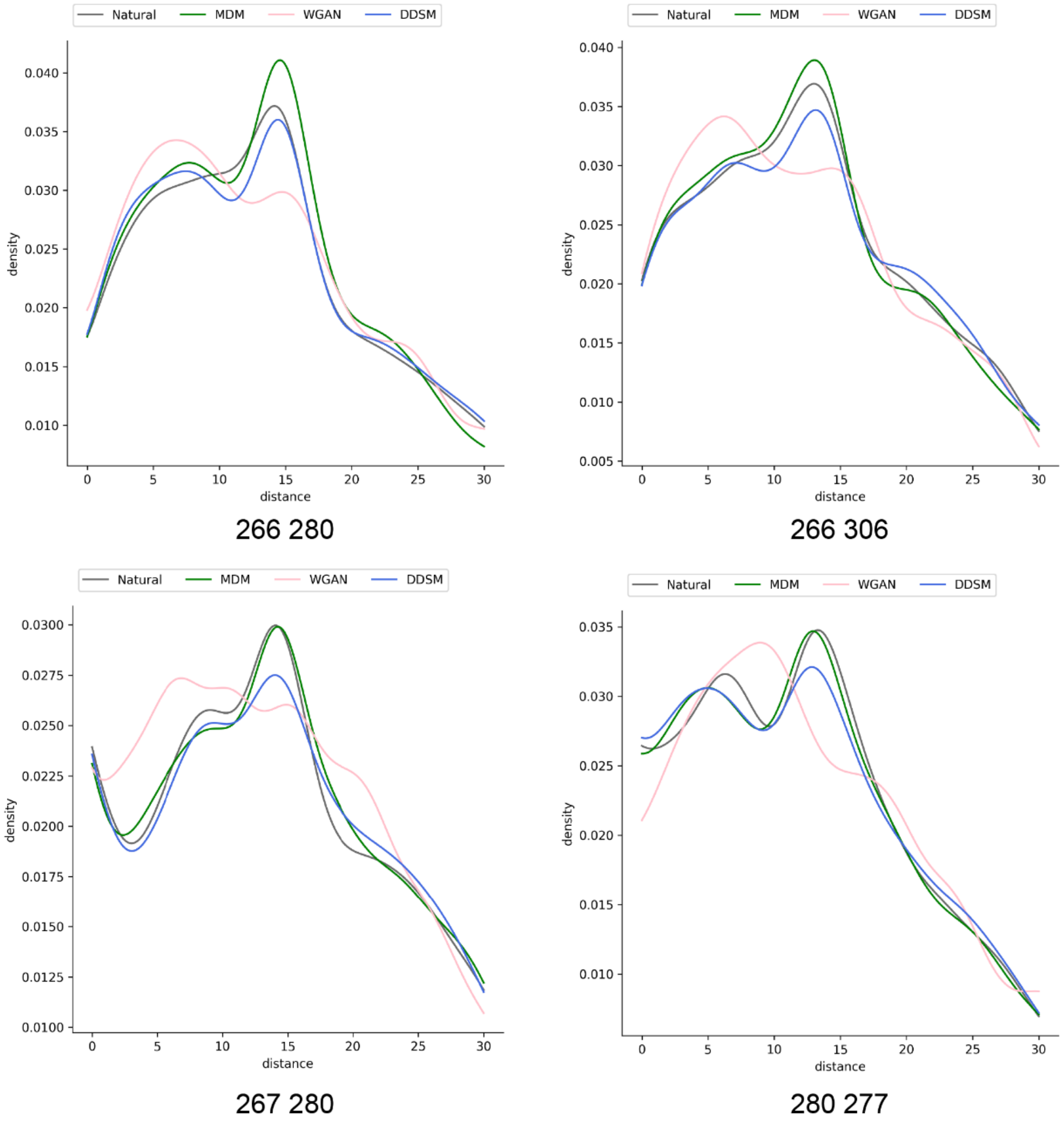

Figure S7. The detailed distribution of inter-motif distances in sequences generated by the model (as described in the main text), each subfigure represents a cell (motif pair) from the heatmap in Figure S6. For each sequence, we fitted a smooth density function to the inter-motif distances using a Gaussian distribution kernel from scipy, with the covariance factor set to 0.15.
